# Analysis of the Clinical Advancements for *BRCA*-Related Malignancies Highlights the Lack of Treatment Evidence for *BRCA*-Positive Male Breast Cancer

**DOI:** 10.3390/cancers14133175

**Published:** 2022-06-28

**Authors:** Dylan P. McClurg, Gordan Urquhart, Trevor McGoldrick, Subarnarekha Chatterji, Zosia Miedzybrodzka, Valerie Speirs, Beatrix Elsberger

**Affiliations:** 1 School of Medicine, Medical Sciences and Nutrition, University of Aberdeen, Foresterhill, Aberdeen AB25 2ZD, UK; mcclurgd01@gmail.com (D.P.M.); s.chatterji.19@abdn.ac.uk (S.C.); zosia@abdn.ac.uk (Z.M.); 2 Aberdeen Royal Infirmary, Department of Oncology, Foresterhill Road, Aberdeen AB25 2ZN, UK; gordan.urquhart@nhs.scot (G.U.); trevor.mcgoldrick@nhs.scot (T.M.); 3 Aberdeen Royal Infirmary, Breast Unit, Foresterhill Road, Aberdeen AB25 2ZN, UK

**Keywords:** male breast cancer, *BRCA*, clinical management, PARP inhibitors

## Abstract

**Simple Summary:**

Male breast cancer (MBC) is an orphan disease that is on the rise but remains understudied. Mutations in genes sensitive to DNA damage response, *BRCA1* and *BRCA2*, are strongly implicated in MBC development. Evidence-based guidance for the treatment of MBC that have *BRCA* mutations is lacking with most published data arising from retrospective or case studies with small patient cohorts. Here, we review the lack of treatment evidence for *BRCA*-related MBC. We also highlight the impact of poly(ADP-ribose) polymerase (PARP) inhibitors which are used in the clinical management of *BRCA*-related female breast cancer and prostate cancer. In turn, we demonstrate the requirement for national and global collaborative efforts to address the striking unmet need for dedicated *BRCA*-related MBC research, including studies to better understand disease trajectory and improve clinical outcomes.

**Abstract:**

Male breast cancer (MBC) is a rare disease that accounts for less than 1% of all breast cancers and male malignancies. Despite recognised clinico-pathological and molecular differences to female breast cancer (FBC), the clinical management of MBC follows established FBC treatment strategies. Loss of function mutations in the DNA damage response genes *BRCA1* and *BRCA2*, have been strongly implicated in the pathogenesis of MBC. While there have been extensive clinical advancements in other *BRCA*-related malignancies, including FBC, improvements in MBC remain stagnant. Here we present a review that highlights the lack of treatment evidence for *BRCA*-related MBC and the required national and global collaborative effort to address this unmet need. In doing so, we summarise the transformative clinical advancements with poly(ADP-ribose) polymerase (PARP) inhibitors in other *BRCA*-related cancers namely, FBC and prostate cancer.

## 1. Introduction

Male breast cancer (MBC) is a rare disease that accounts for less than 1% of all breast cancers and male malignancies [1,2,3,4]. Due to difficulties in achieving sufficient patient numbers, few prospective MBC clinical trials have been conducted and most available data arises from female breast cancer (FBC) trials, small retrospective studies, and case reports/series. As a result, MBC patients generally follow previously established FBC clinical management strategies [5,6]. However, with our increasing knowledge of the differing clinical demographics [7], molecular landscapes [8,9,10,11], histological subtypes [12,13], and prognostic factors between male and FBC [11,14,15], maintaining this ‘one size fits all’ approach is no longer tenable.

Epidemiologically, the incidence of MBC increases with age and typically presents at an advanced stage due to a late presentation at diagnosis and poor MBC awareness within the general population [2,16]. The aetiological factors of MBC remain poorly understood, but a contribution of both hormonal and anthropometric factors that lead to abnormal oestrogen exposure, have been implicated [17]. These include obesity, liver disease, testicular abnormalities, exogenous oestrogen, and Klinefelter syndrome [17]. Like FBC, loss of function mutations in the DNA damage response (DDR) genes that are responsible for genomic stability, *BRCA1* and *BRCA2*, have been heavily implicated in the pathogenesis of MBC. Pathogenic *BRCA* alterations are detected in around 16% of all MBC cases, with 12.5% found in *BRCA2* [18]. Several other genes have been reported to confer a moderate risk of MBC at lower prevalence rates including *CHEK2* (4–8%), *PALB2* (1–2%), and *PTEN* [19,20,21,22,23,24,25,26]. Endeavours to better understand the genetic landscape of MBC have been attempted through genome-wide association and focused gene loci studies. Such studies have identified a number of common polymorphisms that confer MBC risk, including those shared by FBC [27,28,29,30]. Moreover, these susceptibility variants may produce a combinatorial effect on MBC risk in *BRCA*-mutation carriers through a polygenic inheritance model [31].

*BRCA* mutations account for 5–10% of all breast cancers and are responsible for 20–25% of all hereditary breast cancers [32,33]. In addition, driver alterations within *BRCA* provide a substantial risk of developing a number of malignancies other than breast, such as prostate, ovarian, melanoma, and pancreatic [34]. Major efforts have enabled the characterisation of *BRCA* pathogenic gene aberrations within a number of these cancers, including FBC. This has led to the subclassification of patients with preventative risk stratification implications, specific disease courses, and management pathways that include novel targeted therapeutics. However, MBC lags in *BRCA* biomarker-led improvements that influence clinical management, highlighting the lack and need of increased translational research within this area.

Targeted approaches of *BRCA*-mutated neoplasms utilise the homologous recombination repair (HRR) deficiency, and thus the impaired ability to repair double stranded DNA breaks. This confers a greater susceptibility to platinum-based chemotherapy and is the standard treatment for *BRCA*-positive patients in FBC [35,36]. Beyond *BRCA*, an additional important DDR pathway involves the poly(ADP-ribose) polymerase (PARP) enzyme-mediated repair of single-stranded DNA breaks [37,38,39]. Inhibition of PARP function in *BRCA*-related cancers further hinders DNA repair and therefore accelerates tumour cell death. PARP inhibitors (PARPi) have shown significant promise in FBC [40] and castrate resistant prostate cancer [41], and gives credence to their potential therapeutic efficacy in *BRCA*-related MBC.

Despite extensive advancements over the last two decades in the management of FBC patients, and other *BRCA*-related cancers, evidence-based MBC specific guidance is lacking, especially for those with targetable *BRCA* mutations. One bottle neck to this area of research has been the exclusion of male participants in breast cancer trials (although this is slowly changing), and a dearth of studies focused specifically on MBC.

Here we present a review of the lack of evidence available for the treatment of *BRCA*-mutated MBC patients and highlight the substantial gaps in knowledge that are required to better evaluate and understand this unique patient cohort to help inform and improve the current standard of care.

## 2. The Genetic Landscape of MBC

Knowledge of MBC germline mutations have important clinical implications, including the discovery of novel therapeutic targets and specific biomarkers. An overview of high (*BRCA1* and *BRCA2*), moderate (*PALB2, EGFR, CCND1*, and *EMSY*) and low-penetrance (*ESR1*, *TOX3*, and *FGFR2*) germline alterations with clinical translation are summarised below.

### 2.1. BRCA1 and BRCA2

*BRCA1* and *BRCA2* are tumour suppressor genes that are strongly associated with the early development of breast cancers in both, men, and women, but with distinct differences. For example, the lifetime risk of breast cancer development in women carrying *BRCA1/2* is estimated to be 72 and 69%, respectively [42,43,44]. In addition, a *BRCA1*-mutation is associated with the more aggressive molecular phenotype of FBC (e.g., triple receptor–negative, oestrogen receptor (ER) negative, progesterone receptor (PR) negative, and HER2 negative), earlier disease onset, and family history of breast cancer [45]. As a result, women with *BRCA* mutations undergo annual mammographic screening and are recommended to undertake additional adjunct MRI review [46]. Moreover, *BRCA*-positive women are offered risk reduction strategies including prophylactic mastectomy for FBC, and salpingo-oophorectomy to reduce associated ovarian cancer [46].

In contrast to FBC, *BRCA2* mutations confer the greatest risk of MBC development compared to *BRCA1* patients and the general population (*BRCA2*, 8% versus *BRCA1*, 2% versus wild type (WT), 0.1%) [45,47]. Despite the overall absolute risk being lower than their female counterparts, the risk from baseline is substantially greater in males. *BRCA*- associated MBC are usually of a higher grade and commonly present with lymph node metastases [48,49,50,51,52]. Moreover, *BRCA*-associated MBC have been shown to have significantly lower survival rates than *BRCA*-WT patients [53]. In terms of hormone receptor status and HER2 expression, *BRCA1*-mutated MBC are typically ER^+^, PR^+^, and HER^−^, whilst *BRCA2*-positive MBC are ER^−^, PR^−^, and HER2^+^ [50,53,54].

### 2.2. Moderate to Low Penetrance Germline Mutations

Germline mutations in several genes other than *BRCA* have been associated with survival and prognostication in MBC. Reduced survival and aggressive prognostic features are linked to mutated *PIK3CA* and *GATA3* and copy number variations in *PALB2*, *EGFR*, *CCND1* and *EMSY* [8,10,21,55,56,57,58,59,60]. In general, mutations in DNA repair genes were associated with reduced survival, and enrichment of mutations in these genes were also higher in ER positive/HER2 negative MBCs compared to matched FBCs [8]. Single nucleotide polymorphisms such as rs3803662 in the *TOX3* gene and rs2981582 in the *FGFR2* gene have also been associated with an increased risk of MBC development, while the presence of the latter also predicted reduced overall survival [27,61,62].

## 3. Clinical Management of *BRCA*-Related MBC

In general, all MBC patients, dependent on their staging, undergo the same standard of care as per their female counterpart. This includes a modified radical mastectomy and endocrine therapy. Adjuvant chemotherapy and radiotherapy regimens that are offered resemble the treatment strategies of FBC patients. Hormonal therapies available include tamoxifen, which despite a lack of MBC efficacy data, is the adjuvant treatment of choice and is recommended for hormone-receptor positive tumours for a minimum of 5 years [6,63,64]. However, side effects such as weight gain, depression, and impotence have led to high rates of non-compliance and discontinuation in MBC patients [64,65]. In a metastatic setting, aromatase inhibitors are used in tamoxifen resistant cases or in patients who are unsuitable for tamoxifen therapy, however, combination with a gonadotrophin releasing agent, or orchidectomy is required [6,12,66].

In terms of *BRCA*-targeting therapies, encouragingly, MBC patients were included in the OlympiaAD (NCT02000622) [38] and EMBRACA (NCT01945775) [40] phase III trials, which tested the efficacy of Olaparib and Talozoparib, respectively in *BRCA*-related breast cancer. These trials demonstrated 3-month Progression Free Survival (PFS) improvement with PARPi compared to physician’s choice single agent chemotherapy in metastatic *BRCA*-related breast cancer and were subsequently approved as standard therapy in advanced diseased MBC patients. In addition, MBC patients were included in the recent landmark phase III OlympiaA (NCT02032823) [67] trial which demonstrated, for the first-time, improved survival of early breast cancer patients with Olaparib in an adjuvant setting [67]. As a result, the FDA has approved Olaparib while the National Institute for Health and Care Excellence (NICE) is currently evaluating the clinical and cost effectiveness within this clinical context [68].

## 4. *BRCA*-Related MBC Studies

While specific guidelines concerning the management of MBC patients have recently been published [6], men have traditionally been excluded from breast cancer clinical trials. Although this narrative is slowly changing (e.g., the German MBC trial (NCT01638247) that investigated aromatase inhibitors or tamoxifen with gonadotropin-releasing hormone agonist [69]), significant clinical management gaps still remain.

Regarding *BRCA*-positive MBC, there are currently no registered ongoing or recruiting clinical trials. This is not surprising as in addition to frequent exclusion from FBC studies, many attempted clinical trials of MBC have closed due to low participant recruitment (e.g., SWOG-S0511 (NCT00217659)). This phase II trial [70], which evaluated the effects of goserelin and anastrozole in men with recurrent or metastatic breast cancer, was withdrawn due to poor recruitment [70]. In addition, despite the European Organisation for Research and Treatment of Cancer (EORTC) being successful in performing a comprehensive retrospective clinicopathological study of over 1400 MBCs [12], achieving their overarching objective of facilitating MBC clinical trials [5] appears to have been more challenging. Moreover, previous trials that included *BRCA*-positive MBC patients have focussed predominantly on female patients [71]. Despite inclusion, the number of male patients within these studies has been extremely low (*n* ≤ 7) making it impossible to perform subgroup analyses [38,40,67]. As a result, most available data for *BRCA*-positive MBC patients are derived from retrospective studies (Table 1) and case reports (Table 2) [18,48,49,50,52,53,72,73,74,75,76,77,78,79,80,81,82,83,84,85,86,87,88,89,90,91,92,93,94].

The majority of *BRCA*-focused retrospective studies available have provided clinicopathological characterisation of the differing phenotypic features of *BRCA*- positive MBC compared to FBC, and on the whole have described their aggressive nature, differing hormone positivity (ER/PR), familial risk, and associated poorer prognosis [18,48,49,50,52,53,72,73,74,75,76,77,78,79,80,81,82,83,84,85,86,87,88,89,90,91,92,93,94]. This is especially true for *BRCA2*-positive MBC which has been shown to pose a greater risk of earlier aggressive disease onset (age < 60), with associated hypermethylation patterns (e.g., RASSF1) that may serve as prognostic epigenetic markers [49,76,78,90,93]. To date, the largest of these retrospective studies utilised data on 419 MBCs with *BRCA* mutations from an international consortium (Consortium of Investigators of Modifiers) and demonstrated that the majority of MBC cases (89.5%) were *BRCA2* mutation carriers and of high grade [74]. In addition, a study assessing *BRCA*-related cancers in males showed promising results using platinum-based therapy in *BRCA*-related MBC with more than two thirds of patients (*n* = 7) still alive with no disease recurrence after a median follow up of 5.6 years [18]. Nonetheless, these studies are limited by their retrospective nature and, on the most part, low cohort sizes.

In regard to case reports, a number of *BRCA*-positive MBC cases have been reported in the literature (*n* = 13) [73,79,80,81,82,83,84,85,86,87,88,89,91] (Table 2). The majority of these studies (10 of 13) describe accounts of *BRCA*2-mutated MBC cases and highlight the significant familial risk and increased lifetime likelihood of developing MBC or prostate cancer in patients with *BRCA*2 alterations [79,80,82,84,85,86,87,89,91]. For example, a male with prior prostate cancer, who possessed a germline *BRCA2* mutation and a significant family history for breast cancer, was subsequently diagnosed with MBC and underwent curative mastectomy [85]. A further case also reported an account of a *BRCA2*-mutated MBC that received a therapeutic regimen of cyclophosphamide, methotrexate, and 5-fluorouracil, and additional tamoxifen treatment [86]. The patient then went on to develop a new primary cancer of a different hormonal profile which was treated with modified mastectomy [86]. Other studies of particular note include a *BRCA2*-positive patient with metachronous breast and primary lung cancer [79]. Despite a good response from the lung malignancy, the breast cancer was refractive to radiation and platinum-based chemotherapy, and anastrozole [79]. Interestingly, this case was successfully treated with the cyclin dependant kinase inhibitor, Palbociclib, and anti-androgen therapy with a response duration of nearly two years [79]. Palbociclib, and inhibitors of the same class, have shown significant improved outcomes in FBC [95,96]; however, these are yet to be explored in MBC.

## 5. *BRCA* Mutations in Transgender Patients

Transgender persons harbouring *BRCA* mutations and receiving hormonal therapy represent a unique group of patients who also require careful clinical management. Despite an increased incidence of breast cancer in this group [97], there remains no established evidence-based guidance. This has been highlighted in a number of cases, for instance, a recent study describes a *BRCA1*-positive trans female youth receiving hormone therapy to suppress puberty [98]. An additional case involving a transgender woman with a *BRCA1*-alteration went on to develop breast cancer whilst receiving androgen blocking therapy [99]. The patient was subsequently treated with neoadjuvant chemotherapy, mastectomy and adjuvant radiotherapy [99]. With several accounts of breast cancer now noted in transgender women who received feminising hormonal therapy [100], a better understanding of the potential risks of treatment is vital.

## 6. Clinical Trial Led Advancements in Other *BRCA*-Related Cancers

As described above, large randomised clinical trials have led to several advancements in other *BRCA*-related malignancies such as FBC and prostate cancer which are summarised below. These have resulted in the introduction of PARPi into clinical practice and offer a less toxic option than conventional chemotherapeutic agents with significant reductions in quality-of-life deterioration [101].

### 6.1. Female Breast Cancer (FBC)

In FBC, clinical trials investigating PARPi have led to the licencing of both Olaparib and Talozoparib by the US Food and Drug Administration (FDA) and the European Medicine’s Agency (EMA), respectively, for germline *BRCA* (*gBRCA*)-positive advanced breast cancer (Table 3) [38,40,102]

As a result of the randomised, open-label, phase III trial, OlympiAD (NCT02000622) [38], Olaparib was the first PARPi to be approved for *gBRCA*-related advanced FBC [38]. This study evaluated patients who had received two or fewer previous lines of therapy (*n* = 302) using Olaparib monotherapy versus standard chemotherapy. The results demonstrated superior efficacy and tolerability of Olaparib than standard chemotherapy [38]. PFS was also significantly higher in the Olaparib trial arm in comparison to standard chemotherapy (7.0 vs. 4.2 months; hazard ratio (HR), 0.58 (95% confidence interval (CI), 0.43–0.80); *p* < 0.001) (Table 3). In addition, patient objective response rates (ORR) were greater in the PARPi-treated cohort: 59.9 versus 28.8% in those who received chemotherapy [38]. Although further follow up analysis demonstrated no difference in overall survival (OS) between the two treatment groups, it did show that chemotherapy-naive patients who received Olaparib had a longer median OS of 7.9 months, providing a rationale for Olaparib as a future first-line option for *gBRCA* mutated advanced FBC patients in the future [102]. Irrespective of the very small sample size of male participants within this study (Table 3), Olaparib was subsequently approved for both advanced male and FBC by the FDA and EMA, as discussed in Section 3.

Most recently, results of the landmark OlympiA (NCT02032823) [67] trial demonstrated, for the first-time, improved survival of FBC patients with a PARPi in an adjuvant setting [67]. This study included *gBRCA*-positive early breast cancer patients (*n* = 1836) who had completed local treatment and neoadjuvant or adjuvant chemotherapy (Table 3). The Olaparib arm of the study was shown to have superior 3-year distant disease-free survival or death than the placebo (HR 0.57 (95% CI 0.39–0.83); *p* < 0.001) [67] (Table 3). In addition, interim analysis also demonstrated improved 3-year invasive disease–free survival in the therapeutic arm versus the placebo group (HR 0.58 (95% CI 0.41–0.82); *p* < 0.001) [67] (Table 3). Furthermore, no significant adverse events were noted and all safety data were concordant with known side effects of Olaparib [67]. Pivotally, the results of this study have led to FDA approval of Olaparib as an adjuvant treatment for patients with *gBRCA*-mutated HER2-negative high-risk early breast cancer who have already been treated with chemotherapy either before or after surgery. However, this has not been adopted by the EMA or NICE yet. In keeping with the OlympiAD (NCT02000622) study, MBC inclusion within OlympiA (NCT02032823) was limited to just two patients in the Olaparib arm [67] and makes drawing any meaningful conclusions challenging.

The phase III EMBRACA (NCT01945775) [40] trial resulted in the approval of the PARPi, Talazoparid, for the use in *gBRCA*-related, advanced FBC [40]. By comparing the efficacy of Talazoparib (*n* = 287) with a standard single agent of a clinician’s choice (capecitabine, eribulin, vinorelbine, and gemcitabine) (*n* = 144), the PARPi demonstrated a greater median PFS (8.6 versus 5.6 months; HR 0.54 (95% CI 0.41–0.71); *p* < 0.001) (Table 3) and superior ORR (62.2% versus 27.2% (95% CI 2.9–8.8); *p* < 0.001) [40]. Consequently, Talazoparid was also approved for MBC despite the study’s involving only four MBC patients (Table 3).

PARPi have also been studied for their efficacy in combination with standard chemotherapy agents. For example, in the phase III randomised BROCADE (NCT02163694) [103] clinical study, carboplatin/paclitaxel with or without Celiparib was evaluated as a second line treatment in *gBRCA* advanced FBC patients [103]. Results showed a greater PFS (14.5 vs. 12.6 months; HR 0.71 (95% CI 0.57–0.88); *p* = 0.002) (Table 3) in patients treated with Veliparib; however, there was no significant difference in OS between the two trial arms (33.5 versus 28.2 months) [103]. Moreover, the addition of Veliparib to carboplatin and paclitaxel was well tolerated, with low discontinuation rates (<10%) [103].

### 6.2. Prostate Cancer

*BRCA* research-led advances have improved therapeutic options for metastatic and castrate resistant prostate cancer (mCRPC). For example, within the past year, Olaparib was granted FDA approval for mCRPC patients with germline or somatic deleterious HRR gene mutations, including *BRCA1* and *BRCA2*, who progressed following anti-androgen hormonal therapy. The pivotal phase III randomised trial, PROfound (NCT02987543) [41], involved 387 mCRPC patients who were allocated into two cohorts based on DDR defects (cohort A included *BRCA1* and *BRCA2* and *ATM*, while cohort B contained other DDR alterations) [41]. Treatment with Olaparib resulted in a greater median PFS than the anti-androgen control arm (7.4 versus 3.6 months; HR 0.34 (95% CI 0.25–0.47); *p* < 0.0001) [41] (Table 4). Moreover, the ORR was 33 and 2.3% for experimental and control groups, respectively. In addition, *BRCA2*-related patients were found to have a greater PFS benefit after receiving Olaparib when compared to other DDR pathogenic variants (e.g., *ATM*) (Table 4) [41]. Moreover, the PROfound (NCT02987543) [41] study was the first to demonstrate an increase in OS in mCRPC with a PARPi versus physicians choice of second generation-hormonal therapy (19.1 months in cohort A versus 14.7 months in the control arm) (HR 0.69, *p* = 0.02) (Table 4) [104].

Based on the TRITON2 (NCT02952534) [105] trial, the PARPi, Rucaparib, gained accelerated FDA approval for *gBRCA* mCRPC patients progressing after prior androgen hormonal therapy and a taxane chemotherapy [105]. Furthermore, ORRs determined per independent radiology review and investigator assessment, were found to be greatest in those harbouring *BRCA* alterations (43.5% (95% CI 31.0–56.7) and 50.8% (95% CI 38.1–63.4), respectfully) (Table 4) [105]. Full FDA approval will be dependent on the TRITON3 (NCT02975934) [108] phase III randomised control trial which is comparing Rucaparib against physicians’ choice of chemotherapy or second generation hormonal agent in patients who have previously received a hormonal agent but not a taxane drug for mCRPC [108].

Niraparib and Talazoparib PARPi are also being investigated in *BRCA*-related mCRPC. Interim results of the active phase II GALAHAD (NCT02854436) [106] study demonstrated good ORR (41% (95% CI 23.5–61.1)) and PFS (8.2 months (95% CI 5.2–11.1)) with Niraparib in *BRCA*-positive mCRPC patients who have progressed on a second-generation hormonal agent and a taxane chemotherapeutic (Table 4) [106]. In regard to Talazoparib, the phase II TALAPRO-1 (NCT03148795) [107] study showed that patients with *BRCA*-positive mCRPC had superior ORR to the PARPi than other DDR mutations (Table 4) [107]. Both Niraparib and Talazoparib are currently being evaluated in phase III trials for mCRPC.

With promising preclinical support [109,110,111,112], the efficacy of PARPi in prostate cancer is currently being investigated in combination with other agents such as anti-androgens [113,114,115], immunotherapeutics [116], chemotherapy [117], radiotherapy [118], and ATR (ataxia-telangiectasia and Rad3-related) protein inhibitors [119]. Studies involving DDR alterations within their inclusion or primary/secondary outcome measures are outlined in Table 5 and will be described briefly. A total of three trials are currently underway for the evaluation of anti-androgen compounds and PARPi. The phase III PROpel (NCT03732820) [113] trial is exploring Olaparib in combination with abiraterone as first-line therapy in patients with mCRPC [113]. A further phase III study, MAGNITUDE (NCT03748641) [114] is being conducted in both mCRPC patients with and without HRR alterations and the efficacy of niraparib and abiraterone [114]. The benefit of combining Talazoparib and enzalutamide in mCRPC is also being studied in the phase III TALAPRO-2 (NCT03395197) trial [115]. In terms of immunotherapy, one phase I/II study has shown early promise in safety and response profiles when using Durvalumab plus Olaparib in mCRPC (NCT02484404) [116]. Further exploiting the vulnerability of DDR-altered mCRPC to DNA damage, a phase II trial is investigating the impact of the ATRi, Ceralasertib, and Olaparib (NCT03787680) [119]. Other DNA-inhibition strategies that are also being studied include high dose testosterone (NCT03516812) [120]. Ultimately, the amalgamation of PARPi with other anti-cancer compounds could increase the number of DDR-gene mutation positive prostate cancer patients benefiting from PARPi therapy.

## 7. Future Directions in *BRCA*-Related MBC

As highlighted in this review, PARPi are driving transformative improvements in the clinical management of *BRCA*-mutated malignancies. Future directions should aim to evaluate the impact of PARPi, and other targeted approaches, in *BRCA*-positive MBC. This will require the generation of national MBC registries, global collaboration, and pre-clinical studies.

### 7.1. National Registry and Combining Efforts

As an orphan disease, efforts to improve the clinical management of MBC, especially those identified as *BRCA*-positive, will require a global collaborative approach. Impressive efforts by Cardoso et al. [12] have already shown the importance of such collaborations in providing further characterisation of MBC (EORTC International Male Breast Cancer Program). However, *BRCA* MBC focused investigations remain scarce and therefore, consideration should be made on country-specific national registry studies for *BRCA*-mutated male patients (e.g., Scottish/Dutch/French/German national registry studies). This will enable synergistic efforts to carefully design and implement clinical trials with large enough cohorts to prevent early termination and generate enough statistical power to accurately characterise *BRCA*-related MBC, including therapeutic sensitivities. In the long run, this will help improve the clinical management of these patients.

### 7.2. Translational Research

To bridge the gap in the interim of clinical trial development, in vitro and in vivo approaches in *BRCA*-related MBC should also be explored. These could include the generation of patient-derived tumour organoid (PDTO) and patient-derived xenograft (PDX) mouse models to better understand *BRCA*-mutated MBC. Currently, PDTOs do not exist for MBC and are based on FBC organoid derivation, and there are recognised challenges generating organoids from ER-positive disease. In contrast, HER2-positive, and triple negative FBC, have had greater successes [121,122], with the latter phenotype being rarer in MBC [13]. Similar successes have also been achieved with *BRCA*-positive PDX models of FBC. For example, a *BRCA*-mutated (L1780P) PDX model demonstrated a partial response to Olaparib [123].

With coordinated efforts, PDTOs, and PDX models, may be derived from MBCs offering the potential to encompass the clinical diversity of each subtype, including those that are *BRCA*-positive. This will allow further characterisation and exploration of genetic alterations and the identification of corresponding therapeutic sensitivities.

## 8. Conclusions

There is a growing understanding that male and female BCs are distinct diseases with different clinicopathological and molecular characteristics. Despite extensive advancements in other *BRCA*-positive malignancies, there remains a striking unmet need for dedicated research for *BRCA*-related MBC to better understand and optimise clinical management for this subgroup of patients. Such studies are imperative to circumvent the scant information available currently to provide optimal screening and treatment strategies that are tailored for *BRCA*-positive MBC patients.

Due to the rarity of this cancer, dedicated research can only be successful if carried out on a national basis leading into a worldwide collaborative network with established *BRCA*-positive registries in combination with tissue collection for translational research. More imminently, exploration of in vitro and in vivo approaches, such as PDTOs and *PDX* models, may be invaluable in aiding *BRCA*-positive MBC disease characterisation.

## Figures and Tables

**Table 1:** Summary of retrospective studies involving *BRCA*-positive MBC patients.

Author (Year)	Study Population	No. of Patients	Study Objective
Tirkkonen et al. (1999) [94]	MBC patients	25	Somatic genetic alterations in *BRCA2*-associated and sporadic MBC
	*BRCA2*-mutated	5	
Basham et al. (2002) [75]	MBC patients	94	*BRCA1/2*-mutation status and risk of breast cancer in female relatives
	*BRCA1*-mutated	0	
	*BRCA2*-mutated	5	
Ottini et al. (2003) [48]	MBC patients	25	The Characterisation of *BRCA1* and *BRCA2* MBC
	*BRCA1*-mutated	1	
	*BRCA2*-mutated	3	
Kwiatkowska et al. (2003) [76]	MBC patients	43	Investigation of the prognostic value of *BRCA2* status in MBC
	*BRCA2*-mutated	12	
Palli et al. (2007) [93]	MBC patients	99	The association between the *BRCA2* N732H variant and MBC risk
Ottini et al. (2009) [49]	MBC patients	108	Characterisation the clinic-pathological features of *BRCA1/2*- positive MBC
	*BRCA1*-mutated	2	
	*BRCA2*-mutated	8	
Ding et al. (2011) [78]	MBC patients	115	To determine the frequency of pathogenic mutations in *BRCA2* and *PALB2* in MBC cases and to investigate the correlations between mutation status and cancer phenotype
	*BRCA2*-mutated	18	
Ottini et al. (2012) [50]	MBC patients	382	Investigation of the clinical–pathologic features of MBC in association with *BRCA* mutations
	*BRCA1*-mutated	4	
	*BRCA2*-mutated	6	
de Juan et al. (2015) [92]	MBC patients	312	*BRCA1/2* mutations in males with familial breast and ovarian cancer syndrome
	*BRCA1*-mutated	20	
	*BRCA2*-mutated	49	
Gargiulo et al. (2016) [53]	MBC patients	47	Characterisation of MBC, including *BRCA1/2*-mutated patients, and the impact on long-term survival
	*BRCA1*-mutated	1	
	*BRCA2*-mutated	5	
Silvestri et al. (2016) [74]	MBC patients	366 *	To determine if *BRCA1/2* mutation carriers display specific pathologic features and if these differ from FBCs
	*BRCA1*-mutated	40	
	*BRCA2*-mutated	326	
Deb et al. (2017) [90]	MBC patients	60	Investigation of a panel of commonly methylated breast cancer genes in familial MBCs
	*BRCA1*-mutated	3	
	*BRCA2*-mutated	25	
Rizzolo et al. (2018) [77]	MBC patients	69	Gene-specific methylation profiles in *BRCA*-mutation positive and negative MBC
	*BRCA1*-mutated	2	
	*BRCA2*-mutated	8	
Ibrahim et al. (2018) [18]	MBC patients	102	Evaluation of clinical characteristics, pathology findings, treatment selection and survival in *BRCA*-positive males
	*BRCA1*-mutated	0	
	*BRCA2*-mutated	9	
André et al. (2019) [52]	MBC patients	196	Specific biological characteristics and survival in MBC
	*BRCA1*-mutated	0	
	*BRCA2*-mutated	13	
Vietri et al. (2020) [72]	MBC patients	28	Characterisation of *BRCA1/BRCA2* and PALB2 mutations in MBC patients
	*BRCA1*-mutated	2	
	*BRCA2*-mutated	8	

* Original cohort of 419 was restricted to invasive male breast cancer (*n* = 366). **MBC** = male breast cancer.

**Table 2:** Summary of case studies involving *BRCA*-positive MBC patients.

Author (Year)	Study Population	No. of Patients	Study Objective
Savelyeva et al. (1998) [84]	*BRCA2*-mutated MBC	3	Case report describing three brothers with *BRCA2* mutation, two of which developed infiltrating ductal breast cancer
Scheidbach et al. (2000) [87]	*BRCA2*-mutated MBC	1	Describe a case of *BRCA2*-mutation positive MBC
Kwiatkowska et al. (2002) [89]	*BRCA2*-mutated MBC	2	Novel *BRCA2* mutation (frameshift mutation 6621del4 in exon 11) in two male breast cancer cases (father and son) in a Polish family.
Brenner et al. (2004) [86]	*BRCA2*-mutated MBC	1	Highlight a case of *BRCA2*-mutation positive MBC and the implications for screening
Karamanakos et al. (2004) [83]	*BRCA1*-mutated MBC	1	A case of male breast adenocarcinoma in a prostate cancer patient following prolonged anti-androgen monotherapy
Azzouzi et al. (2007) [88]	*BRCA2*-mutated MBC	3	To highlight three *BRCA2*-positive MBC patients who were identified following positive prostate cancer screening
Panchal et al. (2009) [85]	*BRCA2*-mutated MBC	1	A case of *BRCA2*-mutation positive MBC case with a history of prostate cancer
Guaoua et al. (2014) [82]	*BRCA2*-mutated MBC	1	An account of a novel *BRCA2*c.6428C>A p.Ser2143Ter nonsense mutation in a man with familial breast cancer
Benjamin & Riker (2015) [73]	*BRCA1*/HER2-positive MBC	1	To describe a case of a *BRCA1*/HER2 positive MBC
Singer et al. (2015) [80]	*BRCA2*-mutated MBC	1	Highlight the risk of *BRCA2* on multiple cancer risk through a case of prostate and MBC.
Saha et al. (2017) [81]	*BRCA1*-mutated MBC	1	Describe the treatment of MBC by dual HER2 blockade and response prediction using novel optical tomography imaging.
Cheng et al. (2019) [79]	*BRCA2*-mutated MBC	1	To describe an account of metachronous MBC that progressed following radio and chemotherapy which responded to palbociclib, fulvestrant and leuprolide.
Huszno et al. (2019) [91]	*BRCA2*-mutated MBC	1	Clinicopathological analysis of *BRCA2* gene variant, c. 2808_2811delACAA (p. Ala938Profs) in MBC

**MBC**, Male Breast Cancer.

**Table 3:** Summary of clinical trials involving PARPi and *BRCA*-positive FBC and MBC patients.

Phase III Trial (Year)	Trial Arms	Study Population	No. of Patients	Study Result		
			PARPi (F/M)	PFS HR (95%CI)	mPFS (Months)	ORR (%)
**Advanced breast cancer**						
OlympiAD (2017) [38]	Olaparib vs. standard chemotherapy	Patients with <2 lines of previous chemotherapy	205 (200/5)	**0.58 (0.43–0.80);** ***p* < 0.001**	**7.0 vs. 4.2**	**59.9 vs. 28.8**
EMBRACA (2018) [40]	Talazoparib vs. standard single agent of a clinician’s choice *	*gBRCA*-mutated	287 (283/4)	**0.54 (0.41** **–** **0.71);** ***p* < 0.001**	**8.6 vs. 5.6**	**62.2 vs. 27.2**
BROCADE (2020) [103]	Veliparib with carboplatin/paclitaxel vs. carboplatin/paclitaxel alone	*gBRCA*-mutated	337 (333/4)	0.71 (0.57–0.88); *p* = 0.0016	14.5 vs. 12.6	
**Early breast cancer**				**DD or death (99.5%CI)**	**ID or death (99.5%CI)**	
OlympiA (2021) [67]	Olaparib vs. placebo	*gBRCA*-mutated with local treatment and neoadjuvant or adjuvant chemotherapy	921 (919/2)	0.57 (0.39–0.83); *p* < 0.001	0.58 (0.41–0.82); *p* < 0.001	

* Capecitabine, eribulin, vinorelbine, or gemcitabine. Trial results that led to approval are in **Bold. CI**, Confidence Interval; **DD**, Distant disease; **HR**, Hazard Ratio; **ID**, Invasive disease; **mPFS**, median Progression Free Survival; **PARPi**, Poly(ADP-Ribose) Polymerase inhibitor; **PFS**, Progression Free Survival.

**Table 4:** Summary of clinical trials involving PARPi and *BRCA*-positive mCRPC patients.

Trial (Year)	Phase	Trial Arms	Study Population	No. of Patients	Study Result
				PARPi	
PROfound (2020) [41]	III	Olaparib versus standard anti-androgen therapy	Cohort A (*BRCA1*, *BRCA2*, or *ATM* mutation)	162	**rPFS 7.4 m vs. 3.6 m; HR 0.34 (95% CI 0.25–0.47);** ***p* < 0.001**
			Cohort A+ B (Other DDR alterations *)	256	**rPFS 5.8 m vs. 3.5 m; HR 0.49 (0.38–0.63);** ***p* < 0.001**
TRITON2 (2020) [105]	II	Rucaparib	g*BRCA*-mutated mCRPC patients progressing after previous androgen hormonal therapy and a taxane chemotherapy	177	**rORR^a^ *BRCA*-mutated 43.5% (95% CI, 31.0–56.7) and independent investigator ORR 50.8% (95% CI 38.1–63.4)** rORR ^a^ for other HRD-mutation 28.6%; *CHEK2*-mutation 11.1%; *ATM*-mutation 10.5%; *CDK2*-mutation 0%
GALAHAD (2019) [106]	II	Niraparib	mCRPC and biallelic DRD mutated mCRPC patients with disease progression on taxane and androgen receptor-targeted therapy.	81	rORR ^a^ *BRCA*-mutated 41% (95% CI 23.5–61.6); rPFS 8.2 (95% CI 5.2–11.1) rORR ^a^ *BRCA*1/2-WT HRD-mutation 9% (95% CI 1.1–29.2); rPFS 5.3 (95% CI 1.9–5.7)
TALAPRO-1 (2020) [107]	II	Talazoparib	*BRCA*- mutated mCRPC patients with disease progression on taxane and androgen receptor-targeted therapy	46	ORR 43.9%; rPFS 9.3 (95% CI 8.1–13.7)
			*BRCA*-WT mCRPC patients	40	ORR *PALB2*-mutated 33%; rPFS 7.4 (95% CI 2–7.4); *ATM*-mutated 11.8%; rPFS 5.5 95% CI (1.7–8.2)

* Genes included *BRIP1, BARD1, CDK12, CHEK1, CHEK2, FANCL, PALB2, PPP2R2A, RAD51B, RAD51C, RAD51D*, and *RAD54L.* **^a^** Determined by Response Evaluation Criteria in Solid Tumors. Trial results that led to approval are in **Bold. CI**, Confidence Interval; **DDR**, DNA Damage Response; **HR**, Hazard Ratio; **HRD**, Homologous Repair Deficiency; **ORR**, Objective Response Rate; **PARPi**, Poly(ADP-Ribose) Polymerase inhibitor; **rPFS**, radiological Progression Free Survival; **rORR**, radiological Objective Response Rate.

**Table 5:** Summary of clinical trials involving a PARPi in combination with an anti-cancer agent in *BRCA*-positive mCRPC patients.

Trial	Phase	PARPi	Combined Agent
**Anti-androgen therapy**			
PROpel [113]	III	Olaparib	Abiraterone
MAGNITUDE [114]	III	Niraparib	Abiraterone
TALAPRO-2 [115]	III	Talazoparib	Enzalutamide
**Immunotherapy**			
NCT02484404 [116]	I/II	Olaparib	Durvalumab
**ATRi**			
NCT03787680 [119]	II	Olaparib	Ceralasertib
**High dose testosterone**			
NCT03516812 [120]	II	Olaparib	Testosterone enanthate or cypionate

**PARPi**, Poly(ADP-Ribose) Polymerase inhibitor.
